# Xie Zhuo Tiao Zhi formula ameliorates chronic alcohol-induced liver injury in mice

**DOI:** 10.3389/fphar.2024.1363131

**Published:** 2024-04-12

**Authors:** Kaixin Chang, Rui Guo, Wenbo Hu, Xuezhu Wang, Feiwei Cao, Jiannan Qiu, Jiaomei Li, Qiang Han, Zhongyan Du, Xiaobing Dou, Songtao Li

**Affiliations:** ^1^ School of Life Science, Zhejiang Chinese Medical University, Hangzhou, China; ^2^ School of Public Health, Zhejiang Chinese Medical University, Hangzhou, China; ^3^ The First School of Clinical Medicine, Zhejiang Chinese Medical University, Hangzhou, China; ^4^ Key Laboratory of Blood-Stasis-Toxin Syndrome of Zhejiang Province, Zhejiang Engineering Research Center for ‘Preventive Treatment’ Smart Health of Traditional Chinese Medicine, School of Basic Medical Sciences, Zhejiang Chinese Medical University, Hangzhou, China

**Keywords:** Xie Zhuo Tiao Zhi decoction, alcohol-associated liver disease, liver injury, oxidative stress, liver inflammation

## Abstract

This study aimed to evaluate the protective role and potential mechanisms of Xie Zhuo Tiao Zhi decoction (XZTZ) on alcohol-associated liver disease (ALD). XZTZ significantly alleviated alcohol-induced liver dysfunction, based on histological examinations and biochemical parameters after 4-week administration. Mechanically, alcohol-stimulated hepatic oxidative stress was ameliorated by XZTZ, accompanied by the improvement of Nrf2/Keap1 expression and alcohol-activated phosphorylation of pro-inflammatory transcription factors, including JNK, P38, P65, and IκBα, were rescued by XZTZ. In conclusion, XZTZ demonstrates potential in alleviating alcohol-induced liver injury, oxidative stress, and inflammation possibly through modulation of Nrf2/Keap1 and MAPKs/NF-κB signaling pathways, suggesting its potential as a therapeutic option for patients with alcoholic liver disease.

## 1 Introduction

The widespread consumption of alcohol has led to a significant health burden in the form of alcohol-associated liver disease (ALD), impacting a large number of individuals globally (Patel and Flamm, 2023). ALD encompasses a range of metabolic disturbances, from reversible hepatic steatosis to various forms of liver damage such as irreversible alcoholic hepatitis, decompensated cirrhosis, and hepatocellular carcinoma (Dukic et al., 2023). Research indicates that alcohol intake contributes to hepatic oxidative stress, exacerbates liver inflammation, and heightens susceptibility to liver injury in male individuals who consume alcohol (Noonberg et al., 1985).

Despite the development of numerous contemporary pharmaceutical interventions for the prevention and treatment of alcohol-induced liver injury, ALD remains a significant public health concern, primarily attributable to its constrained effectiveness and adverse reactions. To date, minimal progress has been made in the therapeutic approaches for ALD, with abstinence from alcohol being the primary recommended strategy. Consequently, there is an urgent requirement for innovative and more efficacious management strategies.

The traditional herbal formula, rooted in the long history of Chinese medicine and characterized by diverse theories and practices, has been utilized in clinical settings for millennia. Traditional Chinese medicines, particularly compound medicines (known as prescriptions or Fu Fangs in Chinese), have garnered significant global interest for their superior efficacy and safety profiles in the prevention and treatment of various diseases (Cheung, 2011). Current research indicates a growing interest in utilizing traditional herbal formulae as novel therapeutic approaches for treating ALD due to their efficacy, minimal adverse effects, and cost-effectiveness (Chen et al., 2010; Mo et al., 2020; Fang et al., 2023). Among these formulae, maintaining the equilibrium of Yin and Yang is considered a fundamental strategy for restoring health. In contemporary medical terminology, the concept of Yin and Yang can be interpreted as the equilibrium between antioxidants and oxidants (Ou et al., 2003; Szeto and Benzie, 2006).

The Xie Zhuo Tiao Zhi decoction (XZTZ), composed of six herbs: *Shanzha, Heye, Zhiqiao, Baizhu, Fuling*, and *Zexie*, is a modified formulation of Zexie Decoction from the “Golden Chamber • Phlegm and Cough Disease”. Notably, in the Traditional Chinese Medicine Pharmacy of Zhejiang Provincial Hospital of Traditional Chinese Medicine, the XZTZ has been often used in the therapy of metabolic disorders, such as obesity, hyperlipidemia, non-alcoholic fatty liver disease, and other diseases, with the principle of “simultaneously treating symptoms and root causes”, “overall adjustment”, and the advantages of six herbal interactions. Nevertheless, while the pharmacological properties of XZTZ are known, there is a lack of scientific evidence regarding its hepato-protective effects on alcohol-induced liver injury. Thus, the objective of our study was to examine the hepato-protective properties of XZTZ and its potential mechanisms of action in the ALD mice model. This investigation has the potential to offer valuable insights for the prevention and treatment of alcohol-induced liver injury.

## 2 Materials and methods

### 2.1 Drugs and reagents

XZTZ consists of six traditional Chinese medicines, including Shan zha as *Crataegus pinnatifida Bunge [Rosaceae; Crataegi fructus*], He ye as *Nelumbo nucifera Gaertn. [Nelumbonaceae; Nelumbinis folium]*, Zhi qiao as *Citrus × aurantium Engl [Rutaceae; Aurantii fructus]*, Bai zhu as *Atractylodes macrocephala Koidz. [Compositae; Atractylodis macrocephalae rhizoma]*, Fu ling as *Poria cocos (Schw) Wolf [Polyporaceae; Poria]*, Ze xie as *Alisma orientale (Sam.) Juzep. [Alismataceae; Alismatis rhozoma]*. The original botanical drugs were purchased from Traditional Chinese Medicine Pharmacy of Zhejiang Provincial Hospital of Traditional Chinese Medicine. The details of the specific metabolites and contents of the XZTZ dosage are shown in Table 1. The herbs in the XZTZ were soaked in 10 times the amount of distilled water for 1 h, and then extracted by reflux for 3 times. The extract was concentrated to the relative density of 1.05, dried under 60°C, lyophilized into dry powder, and the lyophilized powder was used for subsequent experiments (1 g of lyophilized powder is equal to 12.05 g original herb). Then, it was diluted with normal saline to achieve two concentrations, including 291.2 mg/kg (low dose, XZTZ-L) and 582.4 mg/kg (high dose, XZTZ-H), based on the body surface area of adults (Standard weight 70 kg). Commencing in the second week of the modeling process, the mice were subjected to daily oral gavage administration of XZTZ at the prescribed dosages for a period of 3 weeks. In a previous study by our group, the analysis of the main metabolites of XZTZ using High Performance Liquid Chromatography revealed the following six metabolites in the extract of XZTZ: mannitol (1.23 min) from Shan zha, citric acid (1.7 min) from Shan zha and He ye, quercetin 3-O-β-D-glucuronopyranoside (16.90 min) from He ye, naringin (19.33 min) from Zhi qiao, hesperidin (20.94 min) from Zhi qiao, and alisol A (32.24 min) from Ze xie (Qiu et al., 2023). The chemical composition of the drug in XZTZ complies with the ConPhyMP statement and has been validated for classification at “http://www.plantsoftheworldonline.org”.

**TABLE 1:** The herbs of The Xie Zhuo Tiao Zhi decoction.

Chinese name	Latin name	Medicinal part	Production methods	Place of origin	Batch number	Percentage (%)
Zexie	*Alisma orientale* (Sam.) Juzep	Rhizome	Dried	Sichuan Province	20230601	22.3
Baizhu	*Atractylodes macrocephala* Koidz	Rhizome	Dried	Zhejiang Province	20230401	18.5
Fuling	*Poria cocos* (Schw.)Wolf	Thizome	Dried	Anhui Province	20230801	18.5
Zhiqiao	*Citrus × aurantium* Engl	Fuit rind	Dried	Jiangxi Province	20220909	7.4
Shanzha	*Crataegus pinnatifida* Bunge	Fruit	Dried	Shandong Province	20230101	18.5
Heye	*Nelumbo nucifera* Gaertn	Leafs	Dried	Zhejiang Province	20230401	14.8

Kits for testing the levels of ALT, AST, MDA, SOD, CAT, and GSH-Px were purchased from Nanjing Jiancheng Bioengineering Institute (Nanjing, China). The BCA kit was purchased from the Beyotime Institute of Biotechnology (Jiangsu, China).

### 2.2 Animal experiments

The traditional Lieber-DeCarli alcohol liquid diet and an isocaloric control diet were purchased from Trophic Animal Feed High-tech Co., Ltd (Nantong, China). Twenty male C57BL/6J mice (8 weeks old, 21.00 ± 1.00 g) were provided by the Animal Experiment Center of Zhejiang Chinese Medical University. Animal experiments were reviewed by the Laboratory Animal Management and Ethics Committee (approval number: IACUC-20220321-08). Mice were housed in an environment of 55% ± 5% relative humidity with 23°C ± 2°C and 12 h of light-dark cycle (lights on at 7:30 a.m.). To acclimate the mice, they were continuously free access to regular food and water for 3 days following their acquisition. After 3-day of acclimatization, mice were randomly assigned to four groups for 4 weeks of intervention: a) PF group, fed Lieber-DeCarli liquid diets containing isocaloric maltose dextrin; b) AF group, fed alcohol-containing modified Lieber-DeCarli liquid diets; c) AF with XZTZ-L (AF + XZTZ-L) group; d) AF with XZTZ-H (AF + XZTZ-H) group. After 12 h of fasting, mice were anesthetized with pentobarbital solution (80 mg/kg body weight (Guo et al., 2023)) and euthanized. Blood samples were collected and stored at −80°C until being assayed. Liver tissue was rapidly excised, weighed, and stored at −80°C for further analysis. The Growth parameters of mice among four groups are shown in Table 2.

**TABLE 2:** The Growth parameters of mice.

	PF	AF	AF + XZTZ-L	AF + XZTZ-H
Body weight g)	26.21 ± 0.70	23.56 ± 1.13^a^	24.70 ± 0.77^a^	25.35 ± 0.50^b^
Liver weight g)	0.99 ± 0.06	1.10 ± 0.04^a^	1.07 ± 0.11	1.04 ± 0.03^b^
Liver/body weight ratio	0.038 ± 0.002	0.047 ± 0.002^a^	0.043 ± 0.004	0.041 ± 0.002^b^

Data are expressed as mean ± SD.

^a^ *p* < 0.05 vs*.* PF.

^b^ *p* < 0.05 vs*.* AF.

### 2.3 Histological examination

The liver was fixed in a 4% buffered paraformaldehyde (Biosharp Biotechnology, Shanghai, China) for 24 h. Then it was embedded with an optimum cutting temperature embedding agent and sliced on a cryostat. The 4 µm thick liver sections were stained with H&E.

### 2.4 Immunohistochemistry analysis

The myeloperoxidase (MPO) staining (equivalent to monocytes and neutrophils), the 4 µm thick liver sections were de-paraffinized, and re-hydrated in descending grades of alcohol, followed by heat mediated antigen retrieval procedure. According to the manufacturer’s instruction, sections were incubated in BloxALL solution (Vector Laboratories, Burlingame, CA, United States) to block endogenous peroxidase activity. Then, sections were incubated with anti-MPO antibody (Servicebio, Wuhan, GB12224) overnight at 4°C in a humidified chamber. The secondary antibody was HRP-labeled goat anti-mouse antibodies (Servicebio, Wuhan, G23301) and the DAB chromogenic agent kit (Servicebio, Wuhan, GB12224) was used for histochemical chromography. The MPO staining was evaluated with a light fluorescence microscope at a 20-fold magnification. Three slides were randomly selected for each tissue, and the MPO-positive cells were quantifed by ImageJ software.

### 2.5 IQuantitative real-time polymerase chain reaction (qRT-PCR)

Total RNA was extracted from mice livers using TRIzol reagent (Invitrogen, UK), based on the manufacturer’s protocol. qRT-PCR was performed with the SYBR Green PCR Master Mix (Applied Biosystems, Foster City, CA, United States). Genes levels were normalized to that of 18S and were calculated by the 2^−ΔΔCT^ method. All primers were synthesized by Sangon Biotech Co., Ltd. (Shanghai, China). The sequences of all primers used in this study are listed in Table 3.

**TABLE 3:** The sequences of all primers.

Target genes	Forward primer (5′to 3′)	Reverse primer (5′to 3′)
*18S*	GAA​TGG​GGT​TCA​ACG​GGT​TA	AGG​TCT​GTG​ATG​CCC​TTA​GA
*HO-1*	AAG​CCG​AGA​ATG​CTG​AGT​TCA	GCC​GTG​TAG​ATA​TGG​TAC​AAG​GA
*GCLC*	GGG​GTG​ACG​AGG​TGG​AGT​A	GTT​GGG​GTT​TGT​CCT​CTC​CC
*GPX-1*	AGT​CCA​CCG​TGT​ATG​CCT​TT	GAG​ACG​CGA​CAT​TCT​CAA​TGA
*TNF* *A*	CCC​AGG​GAC​CTC​TCT​CTA​ATC​A	GCT​ACA​GGC​TTG​TCA​CTC​GG
*IL-6*	GAT​GCT​ACC​AAA​CTG​GAT​ATA​ATC	GGT​CCT​TAG​CCA​CTC​CTT​CTG​TG
*IL-1* *B*	TGG​GAT​AGG​GCC​TCT​CTT​GC	CCA​TGG​AAT​CCG​TGT​CTT​CCT
*MCP-1*	AAA​ACA​CGG​GAC​GAG​AAA​CCC	ACG​GGA​ACC​TTT​ATT​AAC​CCC​T

### 2.6 Western blot

Protein concentrations of mice livers were detected using BCA protein assay kits. Samples (approximately 20 μg) were separated by sodium dodecyl sulfate polyacrylamide gel electrophoresis and transferred to polyvinylidene fluoride membranes. The primary antibodies were used as follows: cleaved-Caspase3 (Cell Signaling Techonology, Cat. No. 9664S, rabbit monoclonal, 1:1000), Bcl-2 (Santa Cruz, Cat. No. sc-7382, mouse monoclonal, 1:500), CYP2E1 (Abcam, Cat. No. ab28146, rabbit polyclonal, 1:5000), Nrf2 (Cell Signaling Techonology, Cat. No. 12721S, rabbit monoclonal, 1:1000), Keap1 (Abcam, Cat. No. ab227828, rabbit polyclonal, 1:2000), p-JNK (Cell Signaling Techonology, Cat. No. 9255S, mouse monoclonal, 1:1000), JNK (Cell Signaling Techonology, Cat. No. 9252S, rabbit monoclonal, 1:1000), p-P38 (Cell Signaling Techonology, Cat. No. 4511S, rabbit monoclonal, 1:1000), P38 (Cell Signaling Techonology, Cat. No. 8690S, rabbit monoclonal, 1:1000), p-P65 (Cell Signaling Techonology, Cat. No. 3033S, rabbit monoclonal, 1:1000), P65 (Cell Signaling Techonology, Cat. No. 8242S, rabbit monoclonal, 1:1000), p-IκBα (Cell Signaling Techonology, Cat. No. 2859S, rabbit monoclonal, 1:1000), IκBα (Cell Signaling Techonology, Cat. No. 4814S, mouse monoclonal, 1:1000), LaminB (Cell Signaling Techonology, Cat. No. 17416S, rabbit monoclonal, 1:1000), and β-Actin (Santa Cruz, Cat. No. sc-4778, mouse monoclonal, 1:5000). β-Actin and LaminB were used as the internal control. The secondary antibodies were anti-rabbit (Boster Biological Technology, ba1054, 1:5000) and anti-mouse (Boster Biological Technology, ba1050, 1:5000). Moreover, a nuclear extraction kit (KeyGEN BioTECH, KGP1100) was used for Nrf2 protein. Bands were quantified by the ImageJ software.

### 2.7 Statistics

All data were expressed as the mean ± SD. Statistical analysis was performed using unpaired Student’s t-test with GraphPad Prism 8.02 software (GraphPad Software, San Diego, CA). The *p*-value <0.05 was statistically significant.

## 3 Results

### 3.1 The XZTZ rescues alcohol-induced liver injury in C57BL/6J mice

**FIGURE 1:**
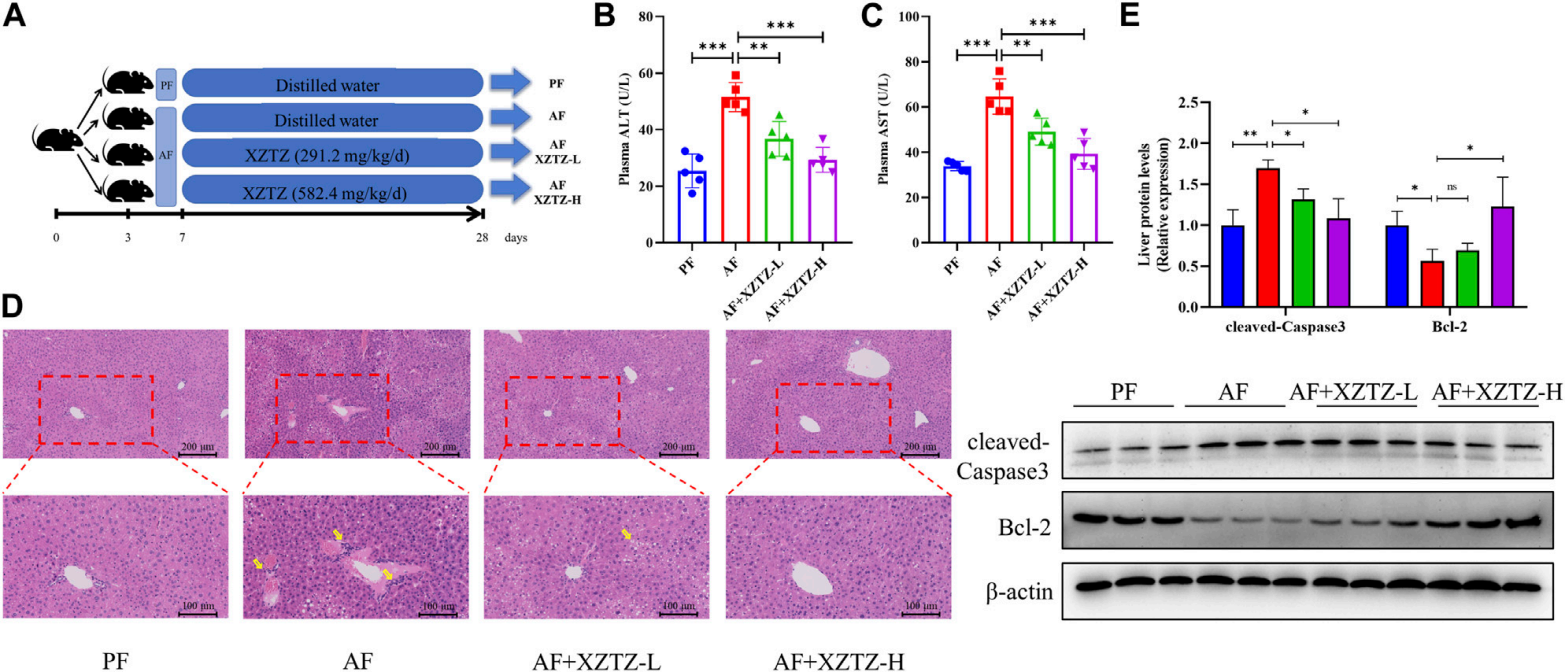
The XZTZ rescues alcohol-induced liver injury in C57BL/6J mice. **(A)** The experimental design graphic; **(B, C)** Serum levels of ALT and AST; **(D)** H&E stained sections of the liver (×200 and ×400); **(E)** Western blot verifying the effects of XZTZ on expression of cleaved-Caspase3 and Bcl-2 with alcohol treatment. ns, not significant; *p < 0.05, **p < 0.01 and ***p < 0.001 indicate statistically significant differences.

In our study, we developed a mouse alcohol-induced liver injury model to evaluate potential recuperative effect of the XZTZ treatment (Figure 1A, Supplementary Figure S1). The increased activities of serum ALT and AST are gold indicators of liver injury. As shown in Figures 1B,C, the activities of serum ALT and AST in the AF group were obviously increased by 2.03-fold and 1.91-fold compared with that in the PF group. The elevations of ALT and AST induced by alcohol were effectively inhibited by the XZTZ, accompanied by a dose-response relationship. We also observed the hepatic pathophysiological changes by H&E staining, and found that XZTZ significantly alleviated alcohol-induced liver injury (Figure 1D). In addition, the elevated hepatic protein level of cleaved-Caspase3 and the decreased hepatic protein level of Bcl-2 induced by alcohol was rescued by the XZTZ (Figure 1E).

### 3.2 The XZTZ alleviates alcohol-induced hepatic oxidative stress in C57BL/6J mice

**FIGURE 2:**
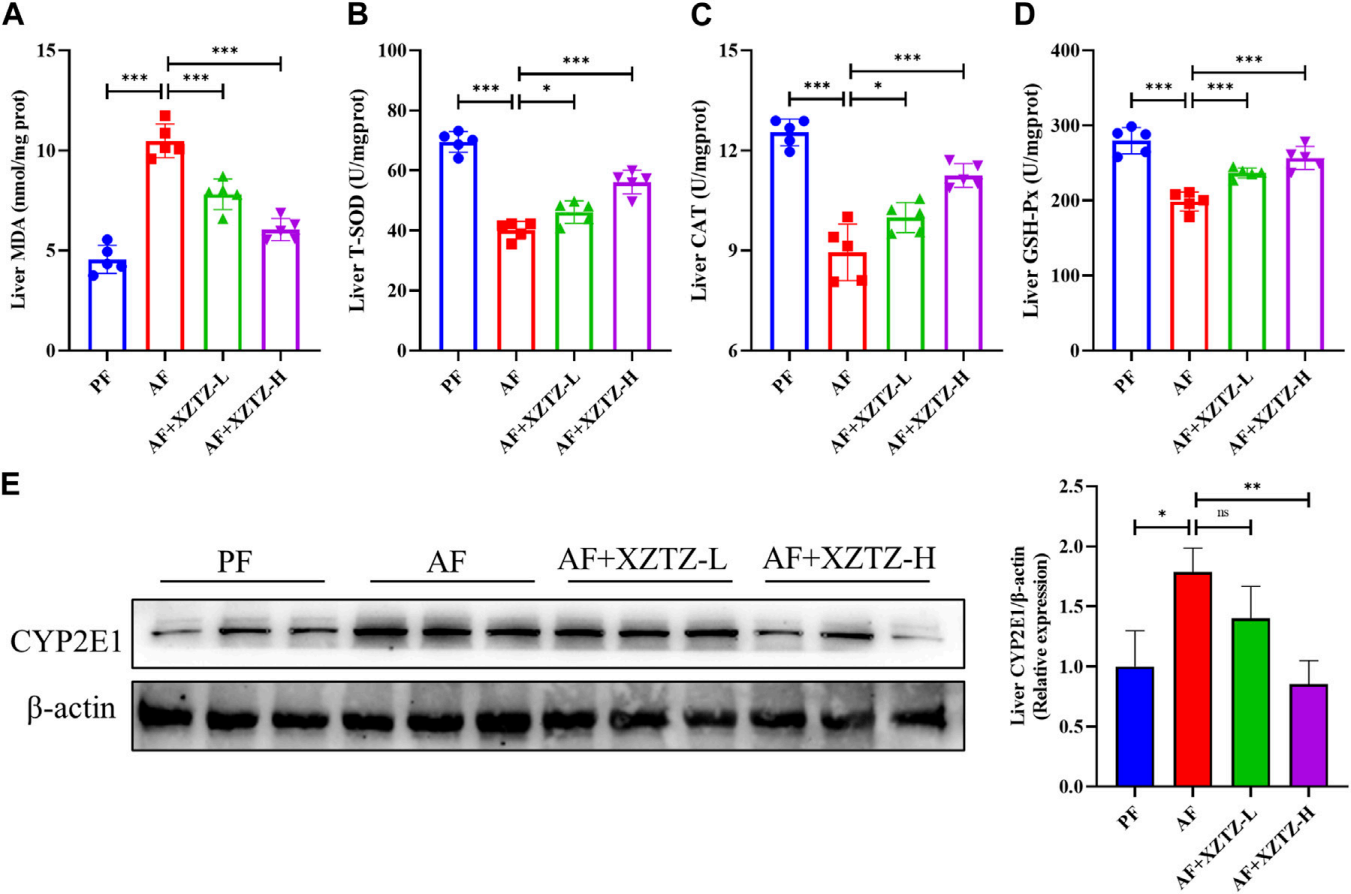
The XZTZ alleviates alcohol-induced hepatic oxidative stress in C57BL/6J mice. **(A)** The level of MDA in mice livers; **(B–D)** The activities of SOD, CAT, and GSH-Px in mice livers; **(E)** The protein level of CYP2E1 in the liver of mice. ns, not significant; *p < 0.05, **p < 0.01 and ***p < 0.001 indicate statistically significant differences.

Oxidative stress is an essential pathological mechanism of alcohol-induced liver injury. Hence, we measured the levels of oxidative stress products and the activities of antioxidant enzymes in alcohol-fed mice livers. As shown in Figure 2A, the hepatic level of MDA was increased in the AF group compared with that in the PF group, while the intervention of the XZTZ significantly reversed the MDA increase induced by alcohol. Also, the activities of SOD, CAT, and GSH-Px were disrupted by alcohol, while the intervention of the XZTZ clearly rescued the activities of the above antioxidant enzymes (Figures 2B–D). Furthermore, CYP2E1 was analyzed in mice model induced by alcohol. Compared with that in the PF group, the expression of CYP2E1 in the liver tissue of mice was significantly increased in the AF group, while the XZTZ treatment decreased its expression (Figure 2E).

### 3.3 The XZTZ inhibits hepatic oxidative stress via activating Nrf2-Keap1 pathway

**FIGURE 3:**
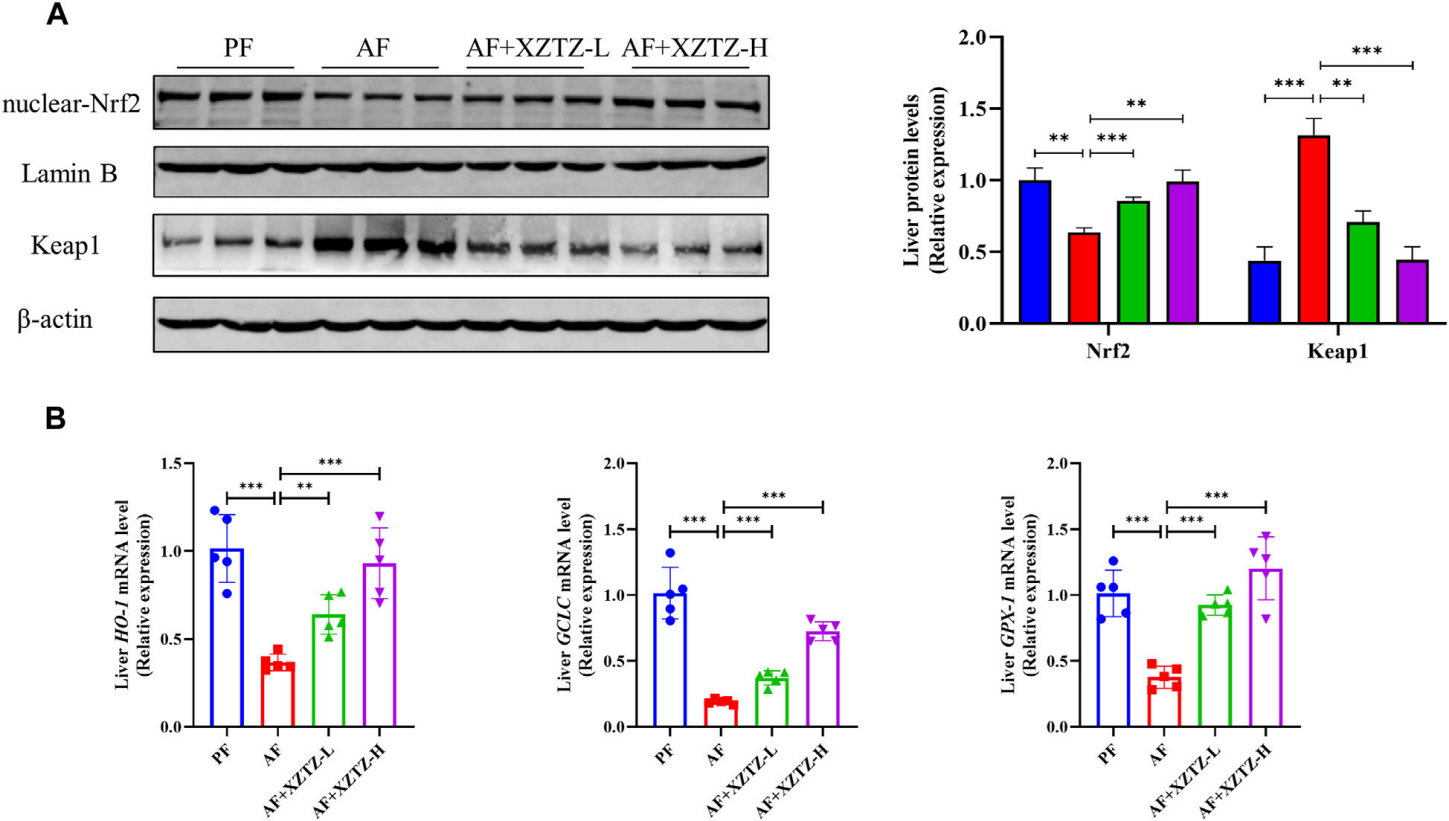
The XZTZ inhibits hepatic oxidative stress via activating the Nrf2-Keap1 pathway. **(A)** Western blot verifying the nuclear protein level of Nrf2 and the total protein level of Keap1. LaminB is used as an internal control for nuclear protein and β-actin is used as an internal control for total protein; **(B)** qRT-PCR verifying the expression levels of *HO-1, GCLC*, and *GPX-1* mRNAs. ns, not significant; *p < 0.05, **p < 0.01 and ***p < 0.001 indicate statistically significant differences.

To further discover the molecular mechanism of the XZTZ on inhibiting oxidative stress, the nuclear protein level of Nrf2 and the total protein level of Keap1 were measured by Western blot. As shown in Figure 3A, compared with the PF group, the hepatic protein level of nuclear-Nrf2 was decreased and the hepatic protein level of Keap1 was increased in the AF group, whereas the levels of Nrf2 and Keap1 were obviously reversed by the XZTZ pretreatment. Moreover, we measured the gene expression levels of *HO-1*, *GCLC*, and *GPX-1* by qRT-PCR in mice livers. The result showed that the XZTZ groups had significantly increased *HO-1*, *GCLC*, and *GPX-1* mRNA expressions compared with the AF group (Figure 3B).

### 3.4 The XZTZ improves alcohol-induced liver inflammation in C57BL/6J mice

**FIGURE 4:**
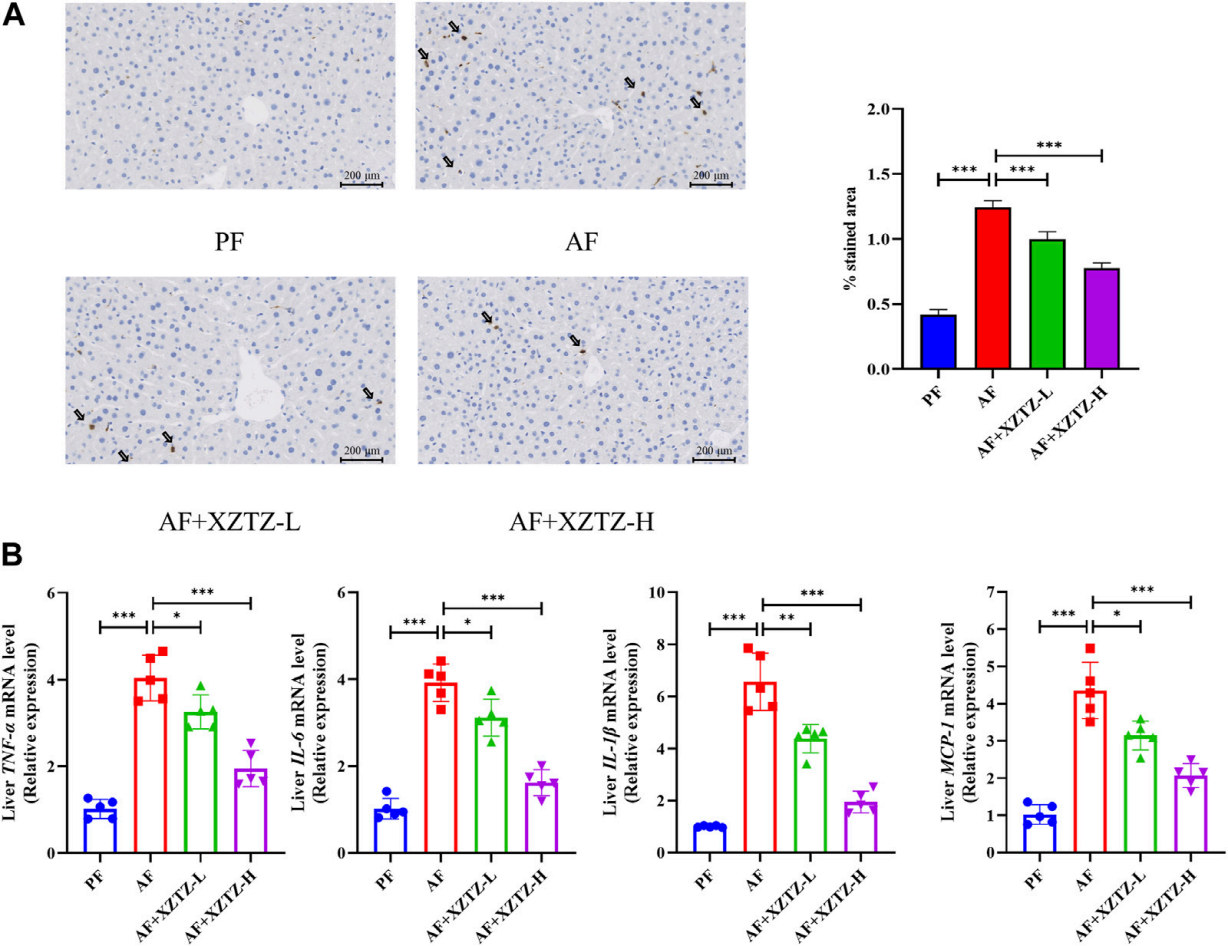
The XZTZ improves alcohol-induced liver inflammation in C57BL/6J mice. **(A)** Representative stainings of MPO-positive cells in mice livers. Scale bars: 200μM; **(B)** Gene expression levels of hepatic *TNFA, IL-6, IL-1B*, and *MCP-1*. *p < 0.05, **p < 0.01 and ***p < 0.001 indicate statistically significant differences.

The inflammation is also a critical pathological process of alcohol-induced liver injury. The MPO staining in our study was performed to visualize monocytes and neutrophils in the different groups. As shown in Figure 4A, there was a robust increase of MPO-positive cells in the AF group compared to other groups, while MPO-positive cells in XZTZ groups were significantly increased compared to the PF group. Additionally, pro-inflammatory cytokines including *TNFA*, *IL-6, IL-1B*, and *MCP-1* in mice livers were significantly increased in the AF group, while the XZTZ supplementation reduced their expressions (Figure 4B).

### 3.5 The XZTZ eases hepatic inflammatory response via MAPKs/NF-kB pathways

**FIGURE 5:**
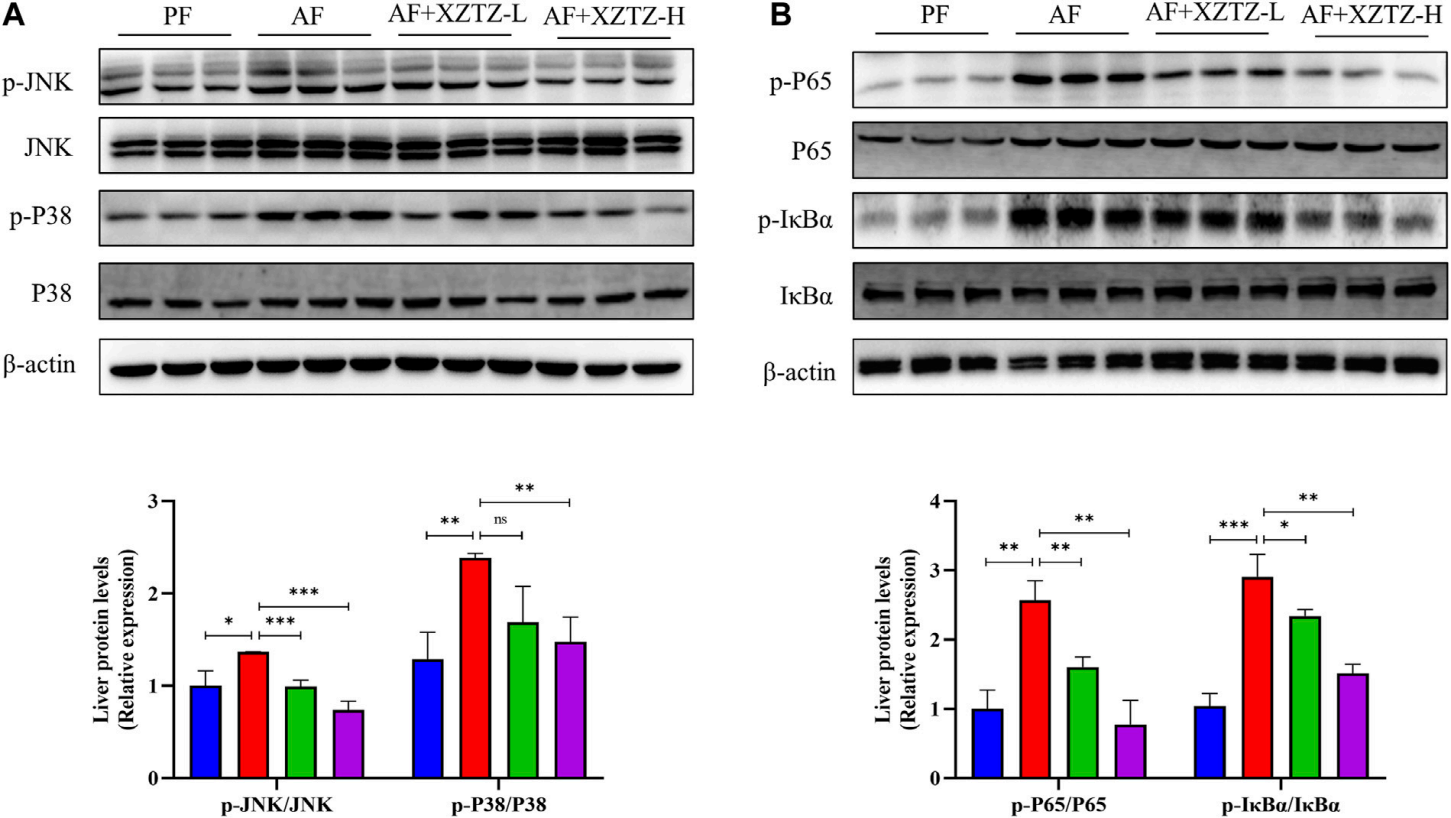
The XZTZ eases hepatic inflammatory response via MAPKs/NF-kB pathways. **(A)** The expression of hepatic p-JNK/JNK and p-P38/P38; **(B)** The expression of hepatic p-P65/P65 and p-IκBα/IκBα. ns, not significant; *p < 0.05 and **p < 0.01 indicate statistically significant differences.

To further explore the molecular mechanism of the XZTZ on alleviating inflammatory response, the expression of proteins involved in MAPKs and NF-κB pathways was measured by Western blot. As shown in Figure 5A, the XZTZ pretreatment significantly reduced alcohol-stimulated both JNK and P38 phosphorylation in a dose-dependent manner. Furthermore, the ratios of p-P65/P65 and p-IκBα/IκBα also decreased clearly after pretreatment with the XZTZ (Figure 5B).

## 4 Discussion

**FIGURE 6:**
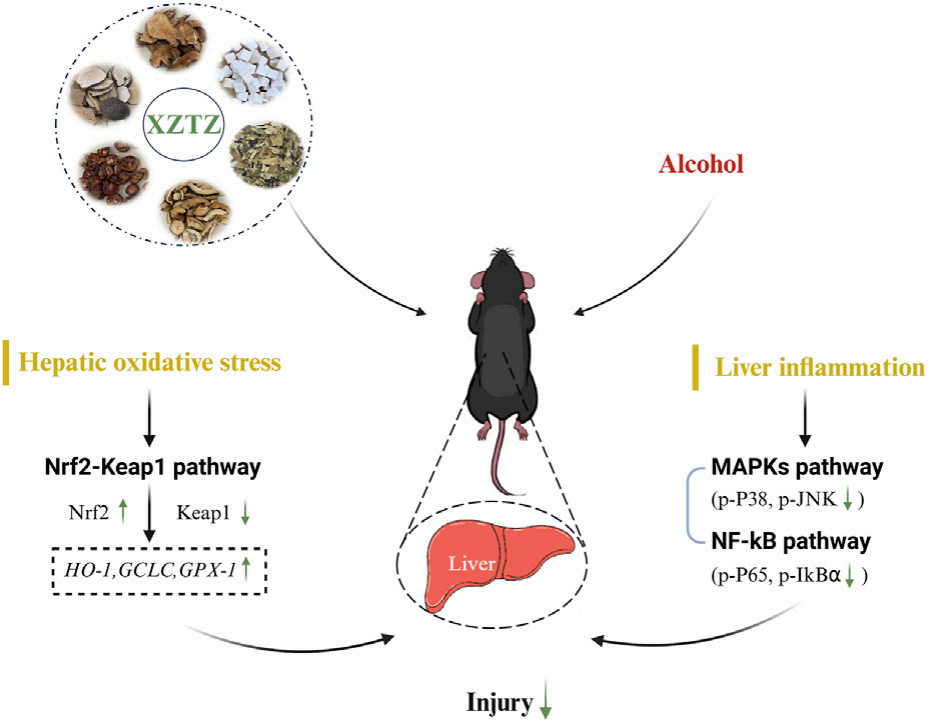
Schematic diagram of our study.

Alcohol consumption is a major global issue for liver damage, with a growing focus on research into preventing and treating alcohol-related liver injury using Chinese herbal medicines (Chen et al., 2010; Feng et al., 2019; Foghis et al., 2023). This study presents the initial evidence that XZTZ, a modified version of the historic prescription ‘Golden Chamber • Phlegm and Cough Disease’, alleviated alcohol-induced liver injury, oxidative stress in the liver, and liver inflammation in C57BL/6J mice in a dose-dependent manner (Figure 6). Using a well-established mice model of ALD, we demonstrated the positive effect of XZTZ in rescuing alcohol-induced liver injury. This was evidenced by decreased ALT and AST activities, reduced hepatic cleaved-Caspase3 expression, elevated hepatic Bcl-2 expression, and H&E staining results (Figure 1). XZTZ also reduced the liver index and aided in the recovery of body weight *in vivo* (Table2).

The contribution of hepatic oxidative stress and liver inflammation to alcohol-induced liver injury is well recognized in the scientific community (Yang et al., 2022; Ding et al., 2023). Many of the herbs in XZTZ exhibit strong antioxidant and anti-inflammatory properties. For instance, *Shanzha* and *Fuling* have demonstrated potential in preventing ALD (Martinez-Rodriguez et al., 2019; Jiang et al., 2022), while *Heye*, *Zhiqiao*, and *Baizhu* have shown protective effects against liver injury by modulating oxidative stress and inflammation levels *in vivo* (Liu et al., 2019; Wu et al., 2020; Guo et al., 2021). The fingerprint analysis of XZTZ has identified six principal compounds: naringin, neo-hesperidin, Atractylenolide III, 23-o-Acetylalisol B, pachymic acid, and ursolic acid. Extensive literature supports the diverse bioactivities and health benefits associated with these compounds, including antioxidant, anti-inflammatory, and hepatoprotective effects (Qiu et al., 2023). MDA, an aldehyde formed during free radical-induced lipid peroxidation, serves as a marker for assessing lipid peroxidation and oxidative stress in the body (Zheng et al., 2019; Yamada et al., 2020). CAT, an essential oxidoreductase, plays a crucial role in breaking down hydrogen peroxide into oxygen and water, thereby safeguarding cells from oxidative stress (Zhao et al., 2019). Our study establishes the hypothesis that the compound medicine XZTZ may offer hepatoprotective effects against ALD by mitigating hepatic oxidative stress and inflammation. Indeed, in our study, ALD mice exhibited elevated MDA levels and reduced activities of SOD, CAT, and GSH-Px, along with decreased CYP2E1 levels in the liver, all of which were reversed upon treatment with XZTZ (Figure 2).

Nrf2, a crucial stress-activated transcription regulator, has been shown to trigger a defense mechanism against hepatic oxidative stress damage. The activation of Nrf2 is typically inhibited by its negative regulator Keap1, but can detach from Keap1 and move into the nucleus during oxidative stress conditions (Jayasuriya et al., 2021). Activation of the Nrf2-Keap1 signaling pathway is a well-established method for reducing oxidative stress-induced liver damage (Choi et al., 2023). Furthermore, in stressful environments, the Nrf2-Keap1 pathway can bind to antioxidant response elements and activate downstream genes like *HO-1*, *GCLC*, and *GPX-1*. The study depicted in Figure 3 demonstrates that pretreatment with XZTZ led to an increase in nuclear Nrf2 protein levels, a decrease in Keap1 protein levels, and an upregulation of downstream genes *HO-1*, *GCLC*, and *GPX-1* in the livers of mice. This suggests that XZTZ may mitigate alcohol-induced hepatic oxidative stress through the Nrf2/Keap1-dependent antioxidant system.

Alcohol-induced acute liver injury triggers the expression of proinflammatory factors like *IL-1B, IL-6,* and *TNFA*, exacerbating organ damage, particularly hepatocyte injury, leading to liver damage (Effenberger et al., 2023). This inflammatory response is recognized as a fundamental mechanism of alcohol-induced liver injury (Wang et al., 2021). Additionally, NF-κB stimulates the production of various proinflammatory factors that play crucial roles in liver pathology (Nowak and Relja, 2020). Alcohol-induced hepatotoxicity can cause the nuclear translocation of NF-κB (p65), leading to the transcription of inflammatory genes such as *IL-1B*, *IL-6*, and *TNFA* (Ghare et al., 2023). MPO staining revealed a significant reduction in MPO-positive cells in the XZTZ groups compared to the AF group. Moreover, XZTZ pretreatment notably decreased the high levels of pro-inflammatory cytokines induced by alcohol in the liver, including *TNFA, IL-6, IL-1B*, and MCP-1, indicating that XZTZ could ameliorate alcohol-induced liver inflammation (Figure 4).

Mechanistically, XZTZ significantly decreased alcohol-induced JNK and p38 phosphorylation in mouse livers (Figure 5A), associated with the MAPKs pathway (Kim and Choi, 2015). Furthermore, XZTZ reduced the ratios of p-P65/P65 and p-IκBα/IκBα, linked to the NF-κB pathway (Lawrence, 2009), in the livers of ALD mice models. In conclusion, XZTZ appears to alleviate alcohol-induced liver inflammation through the MAPKs- and NF-κB-dependent anti-inflammatory mechanisms.

This study is limited by the use of only the classic Lieber-Decarli model to simulate human alcoholic liver disease, which may not fully represent the complexity of the disease. The modified model used is more suitable for studying the early stages of alcoholic liver disease, but further investigation is required to determine the effectiveness of XZTZ in treating late-stage alcoholic liver disease. Additionally, the study did not conduct reverse verification, and the exact mechanism by which XZTZ improves alcoholic liver disease remains unclear, requiring further research. The XZTZ compound used in the experiment contains active ingredients from multiple traditional Chinese medicines, prompting the need to identify the specific ingredients responsible for improving ALD in future research.

## 5 Conclusion

Our research presents evidence suggesting that XZTZ significantly influences alcohol-induced liver injury by mitigating hepatic oxidative stress and decreasing liver inflammation. The antioxidant characteristics of XZTZ could be linked to the Nrf2-Keap1 axis, while its anti-inflammatory properties might be regulated by the MAPKs/NF-κB pathways. These results enhance our comprehension of the therapeutic potential of XZTZ in managing alcohol-induced liver injury within the context of traditional Chinese medicine formulations.

## Data Availability

The original contributions presented in the study are included in the article/Supplementary Material, further inquiries can be directed to the corresponding authors.
