# Supplementary figures and images for: Xie Zhuo Tiao Zhi formula ameliorates chronic alcohol-induced liver injury in mice
Source: Front Pharmacol. 2024 Apr 12;15:1363131. doi: 10.3389/fphar.2024.1363131 (PMC11045942; doi:10.3389/fphar.2024.1363131)

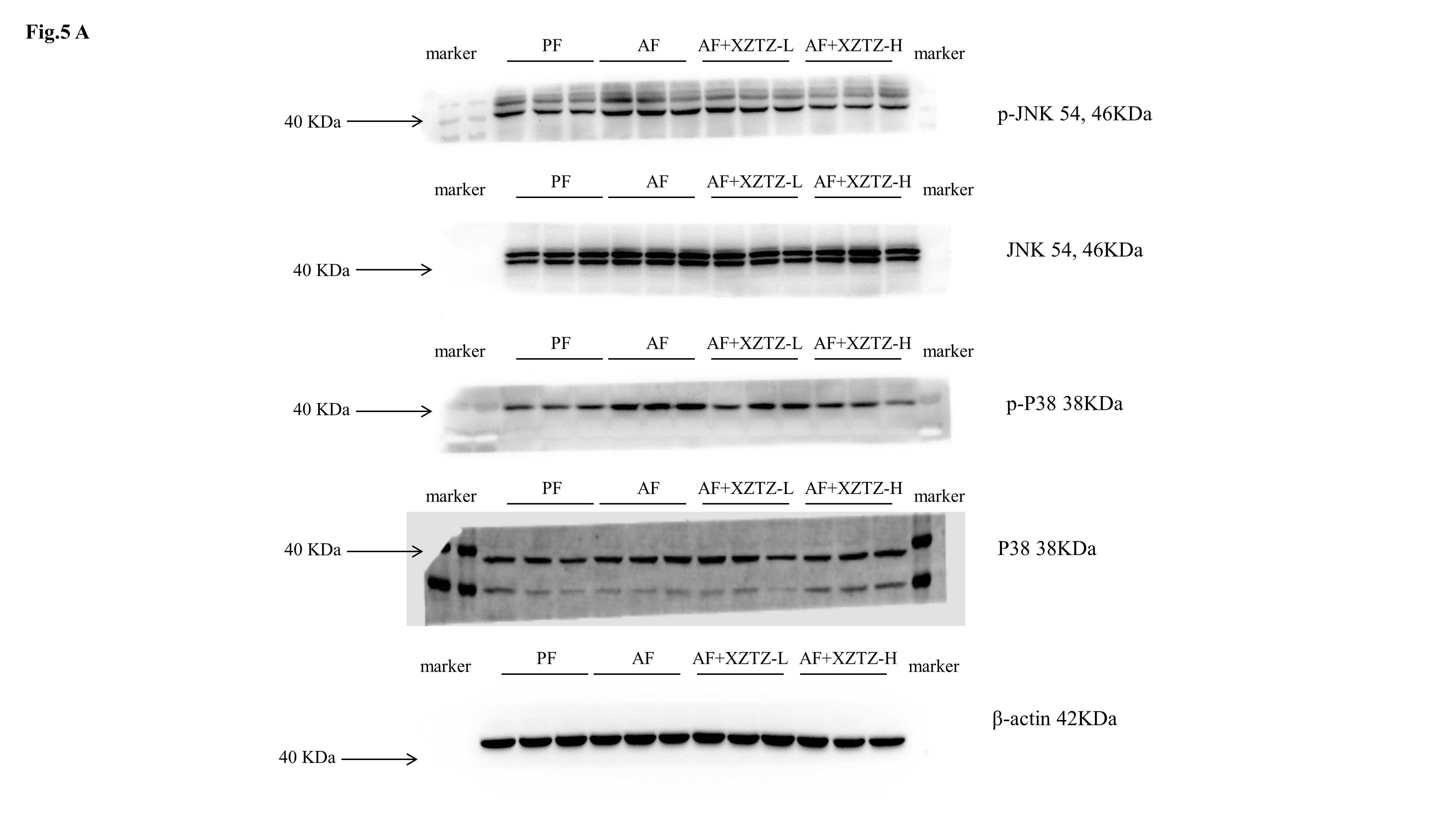

Supplement: Supplementary file 1 [file Image6.TIF]

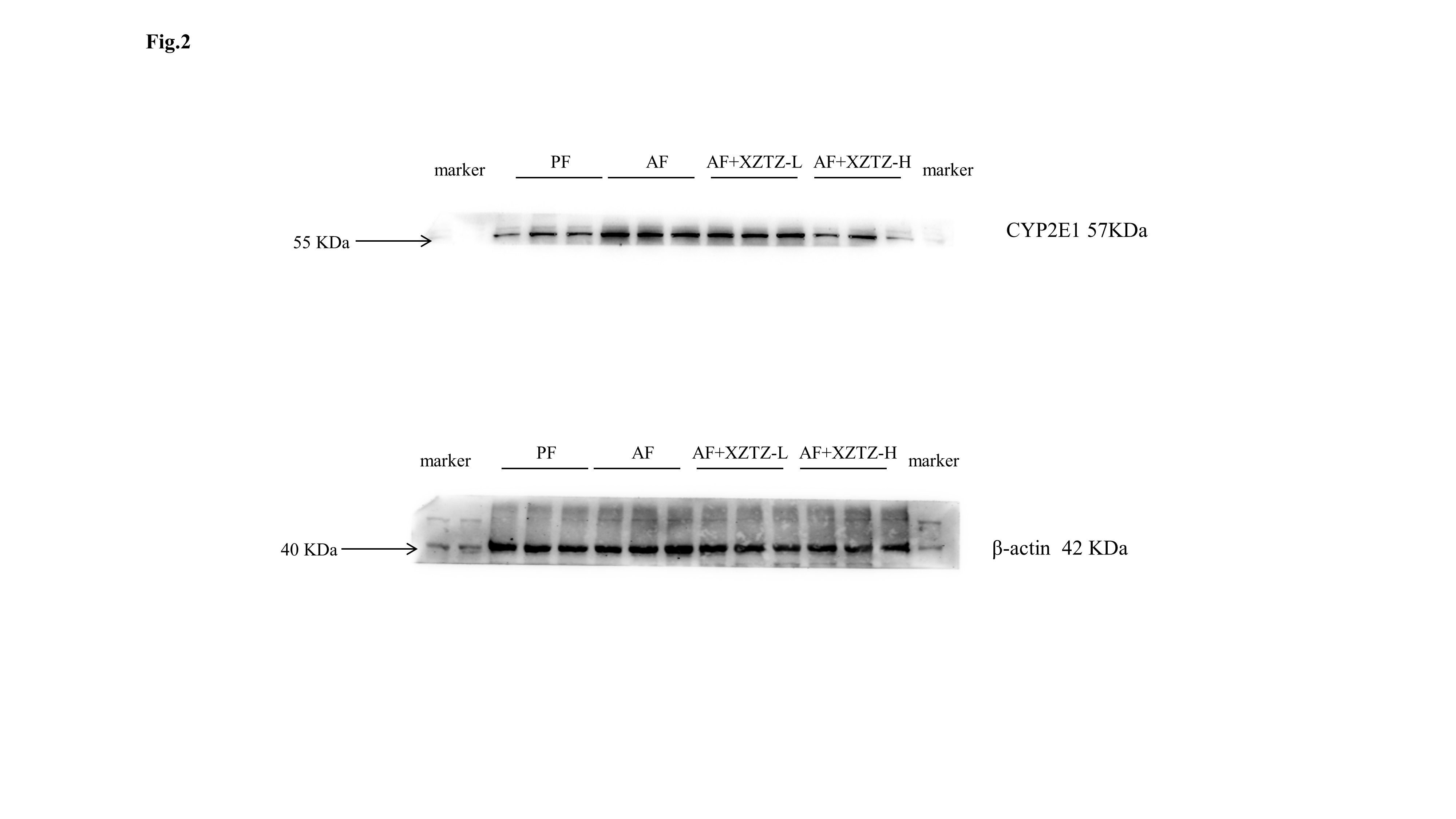

Supplement: Supplementary file 2 [file Image3.TIF]

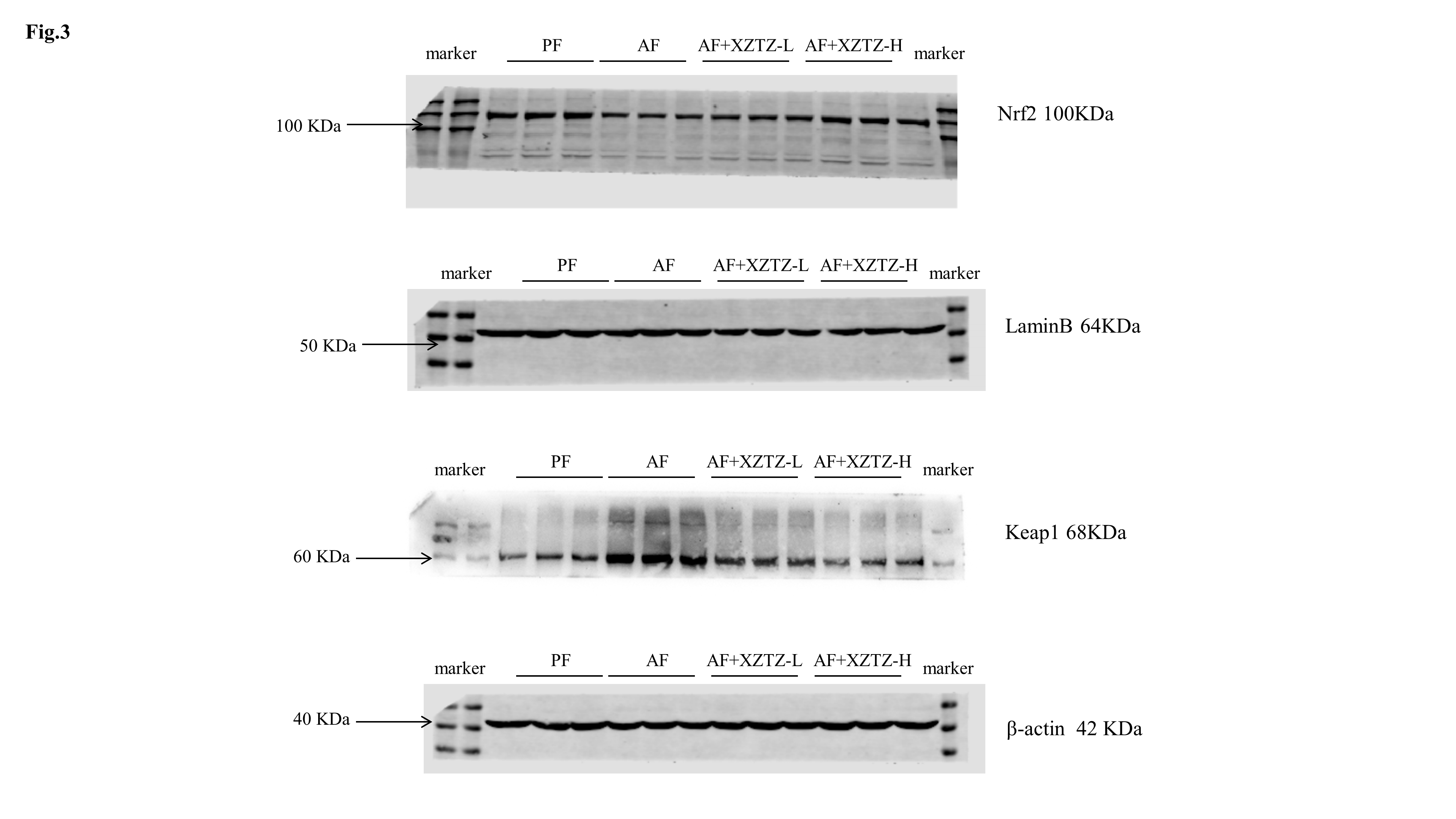

Supplement: Supplementary file 3 [file Image4.TIF]

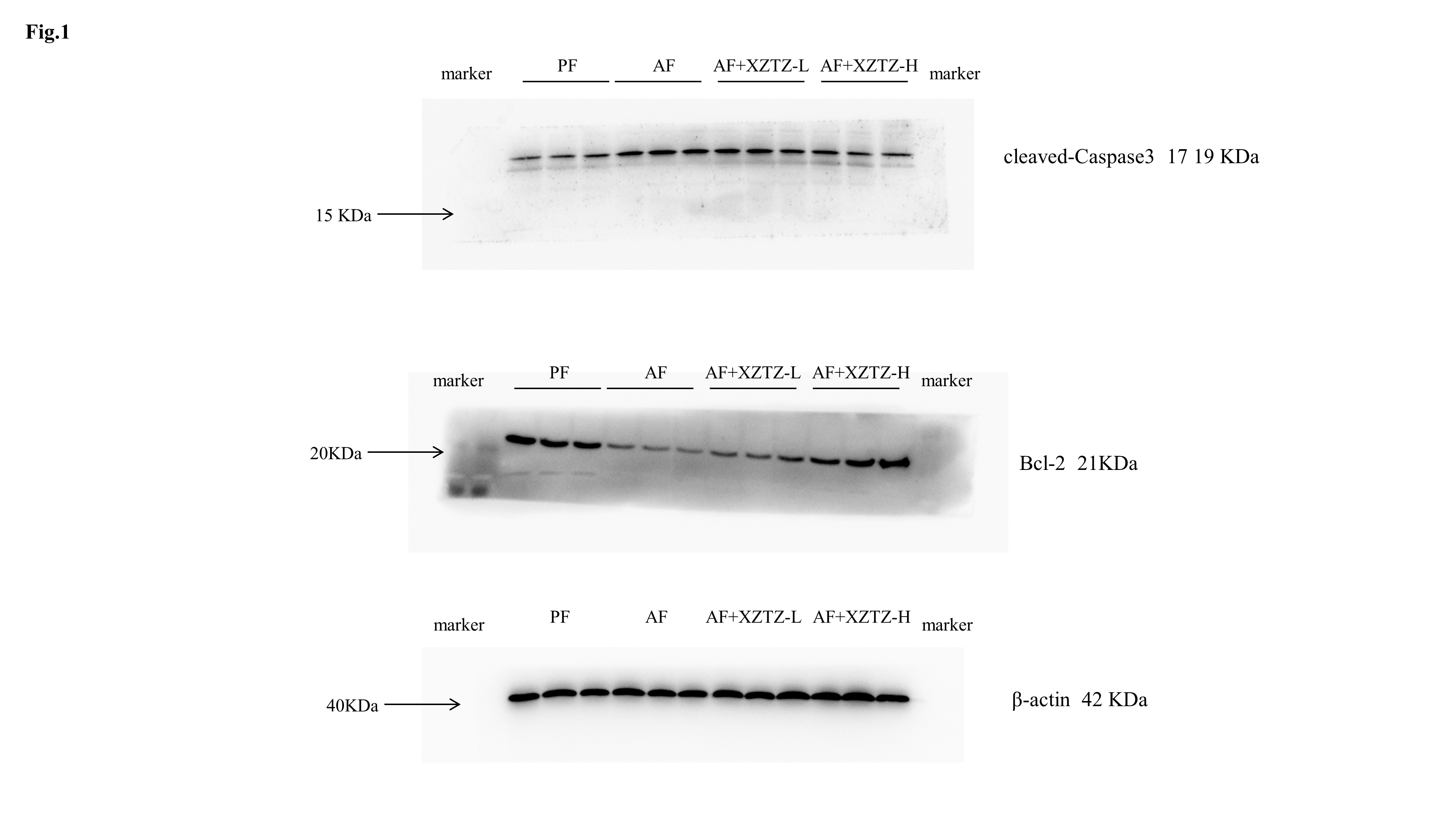

Supplement: Supplementary file 4 [file Image2.TIF]

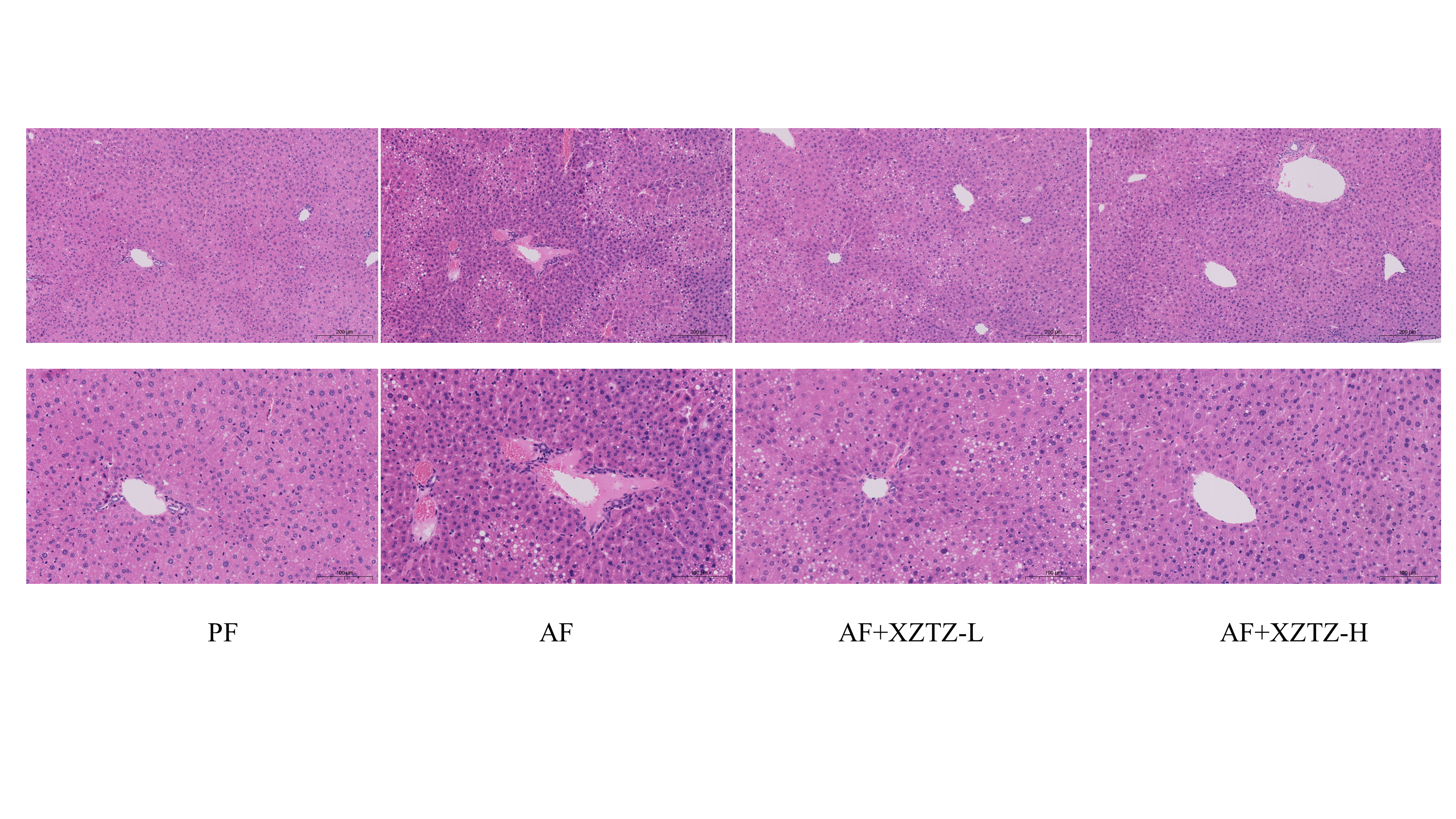

Supplement: Supplementary file 5 [file Image1.TIF]

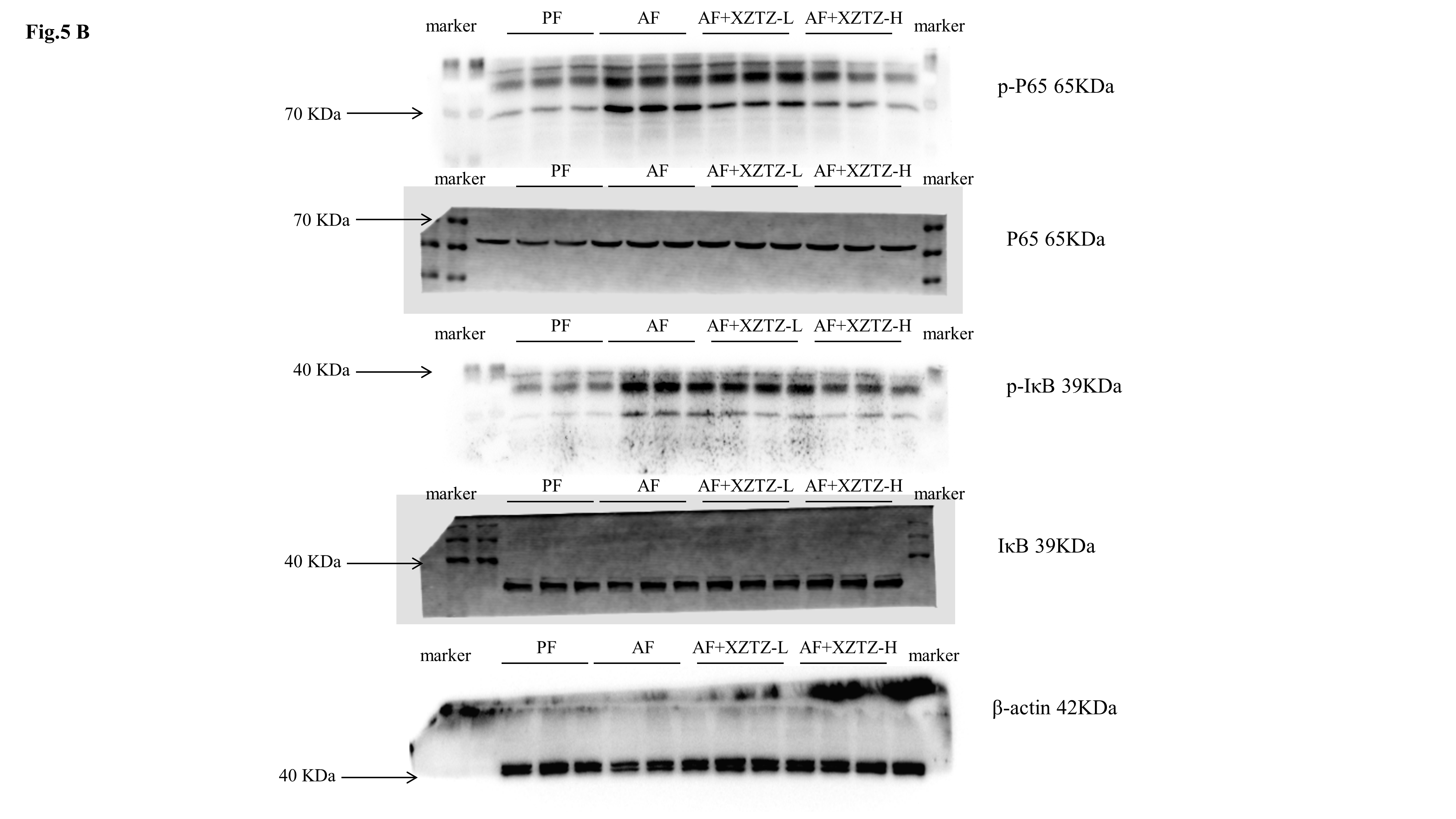

Supplement: Supplementary file 6 [file Image7.TIF]

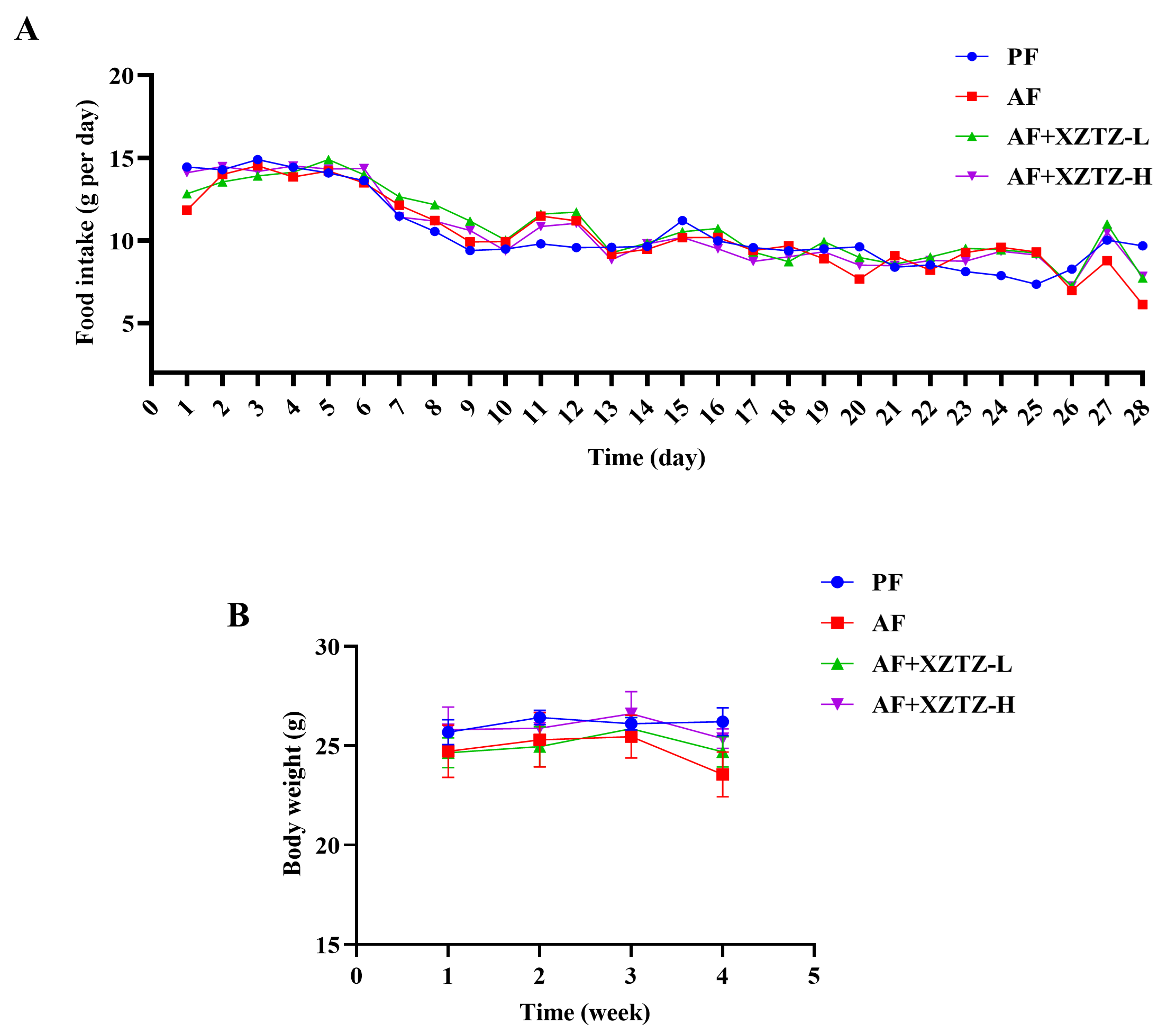

Supplement: Supplementary file 8 [file Image8.TIF]

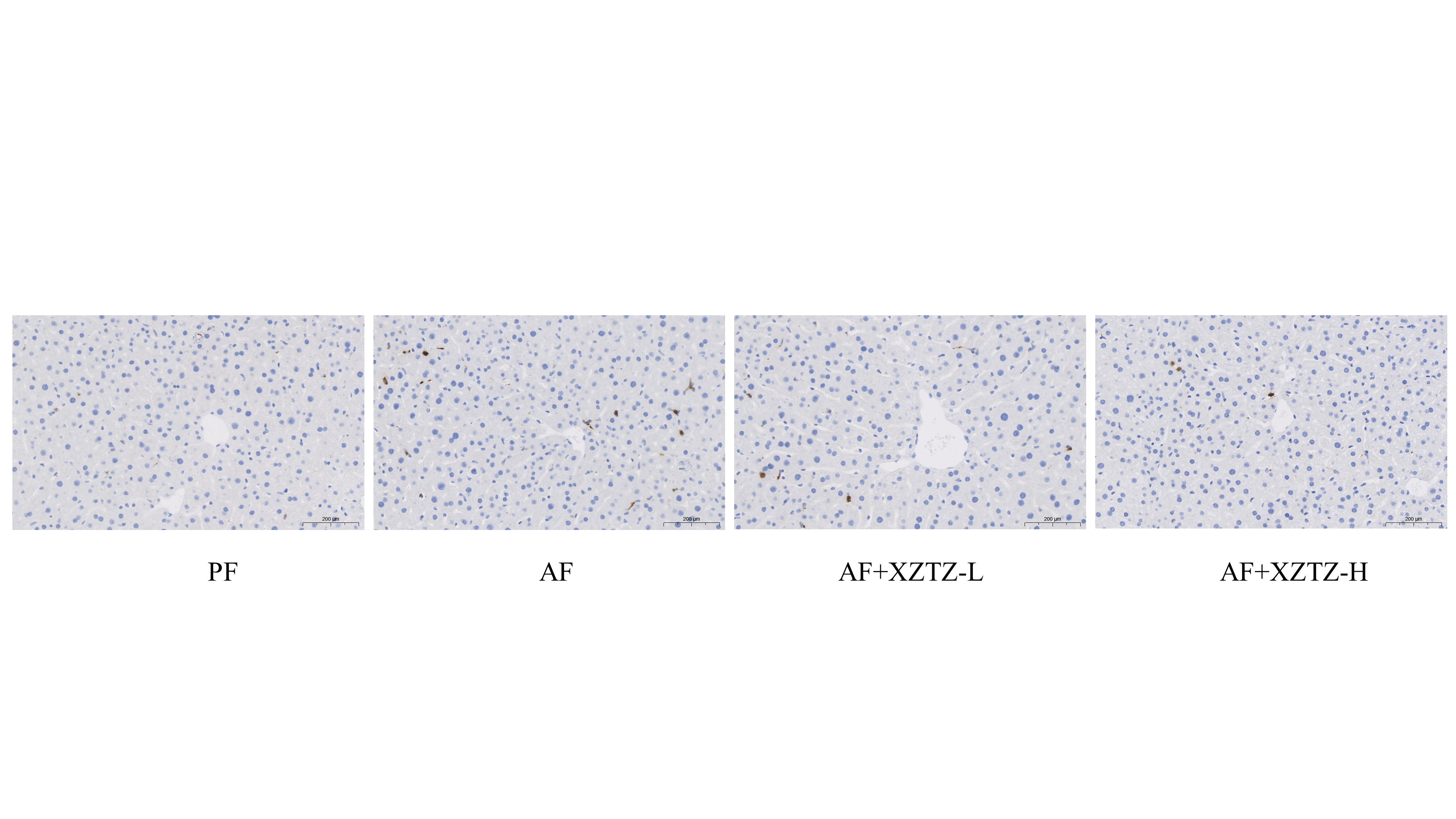

Supplement: Supplementary file 9 [file Image5.TIF]
