# Supplementary material for: Xie Zhuo Tiao Zhi formula ameliorates chronic alcohol-induced liver injury in mice
Source: Front Pharmacol. 2024 Apr 12;15:1363131. doi: 10.3389/fphar.2024.1363131 (PMC11045942; doi:10.3389/fphar.2024.1363131)
Supplement: Supplementary file 7 [file DataSheet2.docx]

**Supplementary Fig. 1**


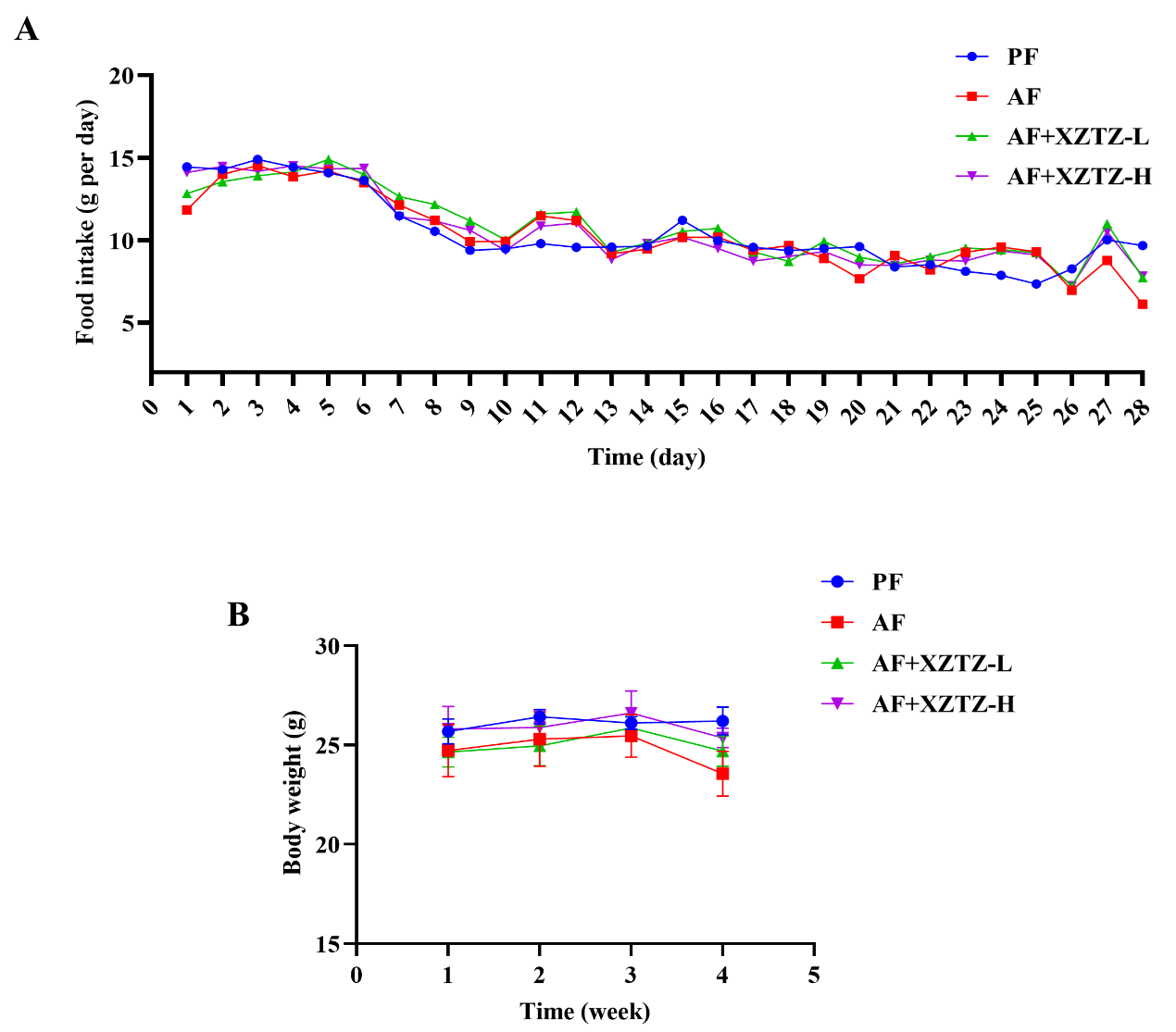


**Supplementary Fig. 1.** Difference in daily food intake and weekly body weight among different groups of C57BL/6J mice. (A) Daily food intake (B) Weekly body weight.
